# Interleukin-6 concentrations in the urine and dipstick analyses were related to bacteriuria but not symptoms in the elderly: a cross sectional study of 421 nursing home residents

**DOI:** 10.1186/1471-2318-14-88

**Published:** 2014-08-12

**Authors:** Pär-Daniel Sundvall, Marie Elm, Peter Ulleryd, Sigvard Mölstad, Nils Rodhe, Lars Jonsson, Bengt Andersson, Mirjana Hahn-Zoric, Ronny Gunnarsson

**Affiliations:** 1 Research and Development Unit, Primary Health Care in Southern Älvsborg County, Sven Eriksonsplatsen 4, SE-503 38 Borås, Sweden; 2 Department of Public Health and Community Medicine/Primary Health Care, Institute of Medicine, Sahlgrenska Academy at the University of Gothenburg, Box 100, SE-405 30 Gothenburg, Sweden; 3 Sandared Primary Health Care Centre, Sandared, Sweden; 4 Health Care Unit Borås Municipality, Våglängdsgatan 21 B, SE-507 41 Borås, Sweden; 5 Department of Infectious Diseases, Institute of Biomedicine, Sahlgrenska Academy at the University of Gothenburg, SE-416 45 Gothenburg, Sweden; 6 Department of Communicable Disease Control, Västra Götalandsregionen, SE-501 82 Borås, Sweden; 7 Department of Clinical Sciences, General Practice, Lund University, CRC, Hus 28, Plan 11, Jan Waldenströms Gata 35, SE-205 02 Malmö, Sweden; 8 Centre for Clinical Research, Dalarna, Falu Vårdcentral, Södra Mariegatan 18, SE-791 70 Falun, Sweden; 9 Department of Public Health and Caring Sciences, Uppsala University, Uppsala, Sweden; 10 Bio Imaging and Laboratory Medicine Unit, Södra Älvsborg Hospital, SE-501 82 Borås, Sweden; 11 Department of Clinical Immunology, Sahlgrenska Academy at the University of Gothenburg, Guldhedsgatan 10A, SE-413 46 Gothenburg, Sweden; 12 Department of Clinical Immunology and Transfusion Medicine, Sahlgrenska Academy at the University of Gothenburg, Guldhedsgatan 10A, SE-413 46 Gothenburg, Sweden; 13 Cairns Clinical School, School of Medicine and Dentistry, James Cook University, Cairns Base Hospital, PO Box 902, Cairns QLD 4870, Australia

**Keywords:** Interleukin-6, Urinary tract infections, Bacteriuria, Homes for the aged, Nursing homes, Dipstick urinalysis, Diagnostic tests

## Abstract

**Background:**

Up to half the residents of nursing homes for the elderly have asymptomatic bacteriuria (ABU), which should not be treated with antibiotics. A complementary test to discriminate between symptomatic urinary tract infections (UTI) and ABU is needed, as diagnostic uncertainty is likely to generate significant antibiotic overtreatment. Previous studies indicate that Interleukin-6 (IL-6) in the urine might be suitable as such a test. The aim of this study was to investigate the association between laboratory findings of bacteriuria, IL-6 in the urine, dipstick urinalysis and newly onset symptoms among residents of nursing homes.

**Methods:**

In this cross sectional study, voided urine specimens for culture, urine dipstick and IL-6 analyses were collected from all residents capable of providing a voided urine sample, regardless of the presence of symptoms. Urine specimens and symptom forms were provided from 421 residents of 22 nursing homes. The following new or increased nonspecific symptoms occurring during the previous month were registered; fatigue, restlessness, confusion, aggressiveness, loss of appetite, frequent falls and not being herself/himself, as well as symptoms from the urinary tract; dysuria, urinary urgency and frequency.

**Results:**

Recent onset of nonspecific symptoms was common among elderly residents of nursing homes (85/421). Urine cultures were positive in 32% (135/421), *Escherichia coli* was by far the most common bacterial finding. Residents without nonspecific symptoms had positive urine cultures as often as those with nonspecific symptoms with a duration of up to one month. Residents with positive urine cultures had higher concentrations of IL-6 in the urine (p < 0.001). However, among residents with positive urine cultures there were no differences in IL-6 concentrations or dipstick findings between those with or without nonspecific symptoms.

**Conclusions:**

Nonspecific symptoms among elderly residents of nursing homes are unlikely to be caused by bacteria in the urine. This study could not establish any clinical value of using dipstick urinalysis or IL-6 in the urine to verify if bacteriuria was linked to nonspecific symptoms.

## Background

The presence of asymptomatic bacteriuria (ABU) among residents of nursing homes for the elderly varies between 25% and 50% for women and 15% and 40% for men [1-3]. There is overwhelming evidence that ABU should not be treated with antibiotics in an adult population except for pregnant women and patients prior to traumatic urologic interventions with mucosal bleeding [4-7]. The high prevalence of ABU makes it difficult to know if a new symptom in a resident with bacteriuria is caused by a urinary tract infection (UTI), or if the bacteria in the urine is only representative of an ABU [3,8-11]. This is especially difficult in the presence of symptoms not specific to the urinary tract such as fatigue, restlessness, confusion, aggressiveness, loss of appetite or frequent falls.

Nonspecific symptoms such as changes in mental status are the most common reasons for suspecting a UTI among residents of nursing homes [12-14]. These symptoms can have many causes besides UTI [15]. There are different opinions on the possible connection between different nonspecific symptoms and UTI [10,16-26]. Nonspecific symptoms and diagnostic uncertainty often lead to antibiotic treatments of dubious value [8,14,27,28]. Urine culture alone seems inappropriate for evaluating symptoms among residents of nursing homes [10]. There are two major possible explanations, either common bacteria in the urine are of little relevance, or a urine culture is insufficient to identify UTI.

With the emergence of multidrug-resistant bacteria and the antimicrobial drug discovery pipeline currently running dry, it is important not to misinterpret bacteriuria as UTI and prescribe antibiotics when it actually represents ABU. Thus, a complementary test to discriminate between symptomatic UTI and ABU is needed [29,30]. The cytokine Interleukin-6 (IL-6) is a mediator of inflammation playing an important role in the acute phase response and immune system regulation [29,31]. The biosynthesis of IL-6 is stimulated by e.g. bacteria [31]. After intravesical inoculation of patients with *E. coli*, all patients secreted IL-6 into the urine, however, serum concentrations of IL-6 did not increase suggesting a dominance of local IL-6 production [32]. A symptomatic lower UTI is assumed associated with more severe inflammation in the bladder compared to an ABU. Previous studies suggested that concentrations of IL-6 in the urine may be valuable in discriminating between ABU and UTI in the elderly, however, this needs evaluation in a larger study among the elderly [9,33].

The aim of this study was to investigate the association between laboratory findings of bacteria in the urine, elevated IL-6 concentrations in the urine, dipstick urinalysis and new or increased symptoms in residents of nursing homes for elderly.

## Methods

During the first three months of 2012, a study protocol was completed and single urine specimens collected from all included residents of 22 nursing homes in south-western Sweden. The attending nurses were provided detailed verbal and written information for the procedure. The study was approved by the Regional ethical review board of Gothenburg University (D-nr 578-11). The data was collected as part of another study of antimicrobial resistance in urinary pathogens among nursing home residents [34].

### Inclusion and exclusion criteria

Residents of the participating nursing homes, regardless of UTI symptoms were invited to participate. Those accepting participation were included if they met the following inclusion criteria:

● Permanent residence in nursing homes for the elderly (regardless of gender)

● Presence at a nursing home for the elderly during the study

● Participation approval

● No indwelling urinary catheter

● Sufficiently continent to leave a voided urinary specimen

● Residents with dementia were included if cooperative when collecting urine samples

● No urostomy

● No regularly clean intermittent catheterisation

● Not terminally ill

● No ongoing peritoneal- or haemodialysis

The following exclusion criterion was used:

● If the resident did not agree to participate or discontinued study participation

### Statement of consent

Residents were informed of the studies verbally and in writing. Informed approval for participation in the studies was collected from decision-capable individuals choosing to participate in the study. However, a considerable number of participants consisted of residents with varying degrees of dementia. If the resident was incapable of understanding information and thereby possessing a reduced decision capability, these residents only participated so long as they did not oppose participation and under the condition that appointed representatives or relatives did not oppose their participation after having partaken of the study information. This procedure was approved by the Regional ethical review board of Gothenburg University.

### Study protocol

In addition to collecting the urine sample, the attending nurse made an entry in the study protocol for each included resident whether having any symptoms, newly onset or increased within the last month and still present when the urine specimen was obtained. Nursing documentation and record keeping was used to obtain information about the presence or absence of symptoms one month prior to inclusion. The following nonspecific symptoms were registered; fatigue, restlessness, confusion, aggressiveness, loss of appetite, frequent falls and not being herself/himself, as well as symptoms from the urinary tract; dysuria, urinary urgency and frequency. It was also registered if the resident had ongoing or previous antibiotic treatment within the last month, diabetes mellitus or dementia.

To avoid presence of symptoms influencing what day the study protocol was completed and urine specimen collected, there was a predetermined date for collection of the urine sample from each included resident.

### Laboratory tests

Personnel at the nursing homes were instructed to collect a mid-stream morning urine sample, or a voided urine specimen with as long a bladder incubation time as possible. Immediately after collecting urine samples, dipstick urinalysis was carried out at the nursing home. Visual reading of the urine dipstick Multistix 5 (Siemens Healthcare Laboratory Diagnostics) was performed for the detection of nitrite and leukocyte esterase. Body temperature was measured by an ear thermometer.

Urine specimens were cultured at the microbiology laboratory at Södra Älvsborg Hospital in Borås, Sweden using clinical routine procedure. The urine specimens were chilled before transport and usually arrived at the laboratory within 24 hours. As in clinical routine, the laboratory was provided information on the outcome of the dipstick urinalysis as well as information on any urinary tract specific UTI symptoms from the attending nurse.

The microbiology laboratory fractionated 10 μl urine on the surfaces of two plates; a cystine-lactose-electrolyte deficient agar (CLED) and a Columbia blood agar base. Plates were incubated overnight (minimum 15 h) at 35-37°C. CLED plates were incubated in air, and Columbia plates were incubated in 5% CO_2_. The latter was further incubated for 24 hours if no growth occurred after the first incubation. Growth of bacteria was considered significant if the number of colony-forming units (CFU)/mL was ≥10^5^. However, at signs of possible UTI such as positive nitrite dipstick, leukocyte esterase dipstick >1, fever, frequency, urgency or dysuria, the cut-off point was ≥10^3^ for patients with growth of *Escherichia coli (E. coli)* and for male patients with *Klebsiella* species (spp.) and *Enterococcus faecalis.* For symptomatic women harbouring the two latter species the cut-off level was ≥10^4^. Nonspecific symptoms did not influence cut-off levels for CFU/mL in the urine cultures.

Measurements of the concentrations of IL-6 in the urine were performed with enzyme-linked immunosorbent assay (ELISA) using a commercial kit (Quantikine HS ELISA, High Sensitivity) [35] according to instructions from the manufacturer (R&D Systems, Abingdon, Oxford, UK) at the clinical immunology laboratory at Sahlgrenska University Hospital in Gothenburg, Sweden. Urine specimens for IL-6 analysis were frozen pending transport to the clinical immunology laboratory.

Concentrations of creatinine in the urine were analysed by the automated general chemistry analyser UniCel® DxC 800 Synchron® Clinical System, according to instructions from the manufacturer (Beckman Coulter), at the clinical chemistry laboratory at Södra Älvsborg Hospital in Borås, Sweden.

### Statistical analysis

The first objective was to clarify whether the concentrations of IL-6 in the urine or urine dipsticks differed between residents with or without bacteriuria. Creatinine adjusted IL-6 was calculated. Concentrations of unadjusted and adjusted IL-6 in the urine and outcome of urine dipstick analyses were compared between residents with positive and negative urine cultures, irrespective of symptoms, using the Mann-Whitney test for IL-6 (due to skewed data) and the Pearson’s chi-square test for urine dipsticks.

The second and third objective was to clarify whether a symptom correlated to bacteriuria or antibiotic usage. The prevalence of bacteriuria or use of antibiotics during the month preceding sampling of urine was compared between residents with or without symptoms using Pearson’s chi-square test. Fisher’s exact test was used in case of small numbers.

The fourth objective was to clarify if the concentrations of IL-6 or outcomes of urine dipsticks differed depending on symptoms in residents with bacteriuria. Concentrations of IL-6 in the urine or outcome of dipstick analyses were compared between bacteriuric residents with or without symptoms using Mann-Whitney’s test for IL-6 (due to skewed data) and Pearson’s chi-square test for dipsticks.

The fifth objective was to correlate factors with symptoms while adjusting for covariates.

A cut-off was used to construct a dichotomous variable covering approximately 20% of the highest IL-6 concentrations (≥5 ng/L). A similar dichotomous variable was constructed for urine dipstick leukocyte esterase where ≥3+ was considered positive. Forward stepwise (conditional) logistic regressions were performed where the condition for entry was 0.050 and for removal 0.10. Variables that served well for the overall prediction were also kept in the model. Zero order correlations between independent variables were checked and correlations >0.6 were not allowed. The independent variables, all but age being dichotomous, were; urine culture, IL-6 in the urine, leukocyte esterase dipstick, nitrite dipstick, antibiotics during the last month, age, gender, and presence of diabetes mellitus or dementia.

IBM SPSS Statistics version 21 was used for statistical analysis.

## Results

### Studied population

**Figure 1:**
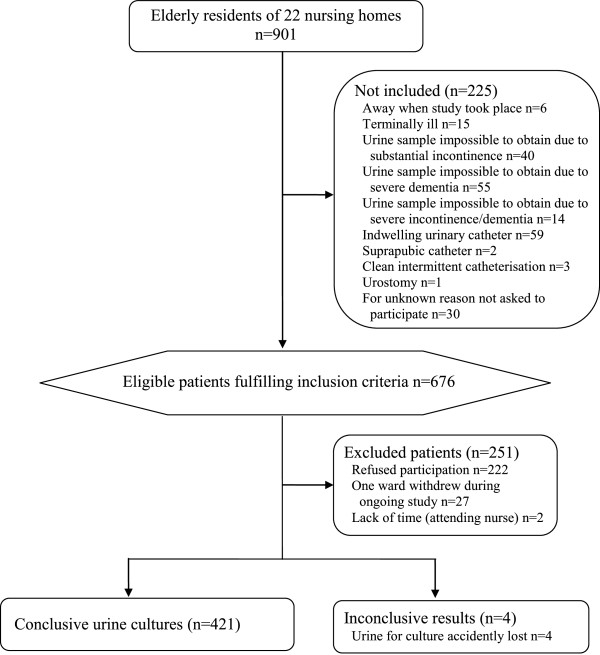
Participant flow chart.

Inclusion criteria were fulfilled by 676 of 901 residents in 22 nursing homes, and 425 (63%) accepted participation (Figure 1). Voided urine specimens and symptom forms were provided from 421 residents, 295 (70%) women and 126 (30%) men. Women (mean 87 years, SD 6.4, range 63-100) were slightly older than men (mean 85 years, SD 7.1, range 65-100) (p = 0.0053).

Among participating residents 56/421 (13%) suffered from diabetes mellitus and 228/421 (54%) had dementia. When urine specimens were collected, 18/421 (4.3%) were undergoing antibiotic treatment. Another 29/421 (6.9%) had no ongoing antibiotic treatment when the urine specimen was collected but had received antibiotics during the previous month. Measure of body temperature was conclusive in 399/421 residents; none of these residents had a body temperature ≥38.0° Celsius.

### Bacterial findings

There was significant growth of potentially pathogenic bacteria in 32% (135/421) of voided urine specimens. *E. coli* was by far the most common finding, present in 81% (109/135) of positive urine cultures. *Klebsiella* spp. were the second most common finding, present in 8.1% (11/135) of positive cultures. *Proteus* spp. were present in 3.0% (4/135) of positive cultures. Other species had very low prevalence’s, ≤1.5% of positive urine cultures for each species.

### IL-6 and creatinine in the urine

Concentrations of IL-6 were analysed in urine specimens from 97% (409/421) of residents. In 2.9% (12/421) of residents, urine samples for IL-6 analyses were accidentally lost, or there was not enough urine for both culture and IL-6 analysis.

Concentration of IL-6 in the urine had a mean of 3.4 ng/L (SD 5.9) and a median of 1.6 ng/L (interquartile range 0.7-4.1, range 0.20-62).

Concentration of creatinine in the urine had a mean of 7.4 mmol/L (SD 4.0). Creatinine adjusted concentration of IL-6 in the urine had a mean of 0.59 ng/mmol creatinine (SD 1.2) and a median of 0.23 ng/mmol creatinine (interquartile range 0.11-0.55, range 0.019-12). Pearson’s correlation coefficient between unadjusted urine IL-6 concentrations and creatinine adjusted IL-6 concentrations was 0.86 (p < 10^-6^).

Urine IL-6 concentrations were ≥5.0 ng/L in 18% (75/409) of residents and creatinine adjusted IL-6 concentrations were ≥0.75 ng/mmol in 18% (75/409) of residents.

### IL-6 concentrations in the urine divided by positive and negative urine cultures

Concentrations of IL-6 in the urine was higher (p = 0.000004) among residents with significant growth of bacteria in the urine; the mean IL-6 concentration was 5.1 ng/L (SD 8.7) and the median IL-6 concentration was 2.5 ng/L (interquartile range 1.0-5.7), compared to residents with negative urine cultures, where the mean IL-6 concentration was 2.6 ng/L (SD 3.6) and the median IL-6 concentration was 1.3 ng/L (interquartile range 0.6-2.8). The same applies for creatinine adjusted IL-6 concentrations (p < 10^-6^).

Similarly residents with positive urine cultures were more likely to have urine IL-6 ≥ 5.0 ng/L (p = 0.000053) and creatinine adjusted IL-6 ≥ 0.75 ng/mmol (p = 0.000001) compared to those with negative urine cultures.

### Dipstick urinalysis

Urine dipsticks were analysed for nitrite and leukocyte esterase in urine specimens from 408/421 residents. Urine dipstick analyses were not performed in 13/421 residents, mostly due to insufficient urine volume. Among all residents, regardless of bacteriuria or not, 26% (106/408) of nitrite dipsticks were positive and 22% (90/408) of leukocyte esterase dipsticks were ≥3 + .

Leukocyte esterase dipsticks ≥3 + were more common (p = <10^-6^) among residents with significant growth of bacteria in the urine; 46% (61/132) versus 11% (29/276) in residents with negative urine cultures. Positive nitrite dipsticks were more common (p = <10^-6^) among residents with positive urine cultures; 64% (84/132) versus 8.0% (22/276) in residents with negative urine cultures.

### Symptoms, bacteriuria and antibiotic treatments

The prevalence of new or increased symptoms, occurring during the last month and still present when urine specimens were obtained are presented in Table 1. There were no significant differences in the proportion of positive urine cultures among those with or without nonspecific symptoms, however there were less positive urine cultures among residents with urinary frequency (Table 1). Residents with some of the symptoms had a higher prevalence of antibiotic treatments during the last month (Table 2).

**Table 1:** Prevalence of symptoms and positive urine cultures

		**Proportion of positive urine cultures among**		
	**Prevalence of symptom**^**1**^	**Residents with symptom**	**Residents without symptom**	**P-value**^**2**^
Fatigue	11% (48/421)	31% (15/48)	32% (120/373)	0.90
Restlessness	5.5% (23/421)	26% (6/23)	32% (129/398)	0.53
Confusion	5.2% (22/421)	14% (3/22)	33% (132/399)	0.057
Aggressiveness	5.0% (21/421)	19% (4/21)	33% (131/400)	0.19
Loss of appetite	5.2% (22/421)	18% (4/22)	33% (131/399)	0.15
Frequent falls	5.2% (22/421)	23% (5/22)	33% (130/399)	0.34
Not being herself/himself	4.3% (18/421)	39% (7/18)	32% (128/403)	0.53
Having any of the above nonspecific symptoms	20% (85/421)	31% (26/85)	32% (109/336)	0.74
Dysuria	2.1% (9/421)	11% (1/9)	33% (134/412)	0.28
Urinary urgency	3.6% (15/421)	33% (5/15)	32% (130/406)	1.0
Urinary frequency	2.4% (10/421)	0% (0/10)	33% (135/411)	**0.035**

^1^Symptoms commencing at any time during the preceding month and still present when sampling urine.

^2^Pearson’s chi-square and when appropriate Fisher’s exact test comparing proportions of positive urine cultures among those with or without symptoms.

**Table 2:** Prevalence of symptoms and antibiotic treatment

		**Proportion of antibiotic treatment**^**2**^**among**		
	**Prevalence of symptom**^**1**^	**Residents with symptom**	**Residents without symptom**	**P-value**^**3**^
Fatigue	11% (48/421)	19% (9/48)	10% (38/373)	0.076
Restlessness	5.5% (23/421)	22% (5/23)	11% (42/398)	0.16
Confusion	5.2% (22/421)	27% (6/22)	10% (41/399)	**0.026**
Aggressiveness	5.0% (21/421)	19% (4/21)	11% (43/400)	0.28
Loss of appetite	5.2% (22/421)	36% (8/22)	10% (39/399)	**0.0013**
Frequent falls	5.2% (22/421)	27% (6/22)	10% (41/399)	**0.026**
Not being herself/himself	4.3% (18/421)	17% (3/18)	11% (44/403)	0.44
Having any of the above nonspecific symptoms	20% (85/421)	19% (16/85)	9.2% (31/336)	**0.012**
Dysuria	2.1% (9/421)	89% (8/9)	9.5% (39/412)	**<10**^**-6**^
Urinary urgency	3.6% (15/421)	53% (8/15)	10% (39/406)	**0.000048**
Urinary frequency	2.4% (10/421)	30% (3/10)	11% (44/411)	0.090

^1^Symptoms commencing at any time during the preceding month and still present when sampling urine.

^2^Antibiotic treatment given at any time during the month preceding sampling of urine.

^3^Pearson’s chi-square and when appropriate Fisher’s exact test comparing proportion of antibiotic treatment among those with or without symptoms.

### IL-6 and dipstick urinalyses in residents with bacteriuria

In residents exclusively with bacteriuria there were no significant differences in concentrations of urine IL-6 when comparing those with or without a new or increased symptom; fatigue (p = 0.24), restlessness (p = 0.40), confusion (p = 0.38), aggressiveness (p = 0.66), loss of appetite (p = 0.27), frequent falls (p = 0.15), not being herself/himself (p = 0.90), having any of the nonspecific symptoms (p = 0.69), dysuria (p = 0.13) and urinary urgency (p = 0.82).

In residents exclusively having bacteriuria there were no significant differences in the proportion of leukocyte esterase dipsticks ≥3+ when comparing those with or without new or increased symptoms; fatigue (p = 0.39), restlessness (p = 1.0), confusion (p = 1.0), aggressiveness (p = 0.62), loss of appetite (p = 1.0), frequent falls (p = 0.60), not being herself/himself (p = 1.0), having any of the nonspecific symptoms (p = 0.68), dysuria (p = 0.46) and urinary urgency (p = 0.34). Similarly there were no significant differences in proportion of positive nitrite dipsticks when comparing those with or without new or increased symptoms.

All patients with urinary frequency had negative urine culture.

### Predictors of symptoms

A positive urine culture was only significant in the model predicting confusion, OR 0.15 (0.033-0.68; p = 0.014). However, it is important to note that the odds ratio was <1, i.e. positive urine cultures were less common among residents with confusion (Table 3). As urine IL-6 > 5 ng/L was also a significant predictor in this regression model for confusion, another regression was made where urine culture and urine IL-6 ≥ 5 ng/L were replaced by a combined dichotomous variable being positive if both IL-6 ≥ 5 ng/L and the urine culture was positive at the same time, or otherwise negative. This combined variable was however not a significant predictor of confusion.

**Table 3:** **Predictors**^**1 **^**of new or increased symptoms commencing at any time during the preceding month and still present when sampling urine**

	**Bacteriuria**^**2**^	**IL-6**^**3**^	**Antibiotics**^**4**^	**Dementia**	**R square**^**1**^
Fatigue^5^	---	---	---	---	---
Restlessness^5^	---	---	---	---	---
Confusion	0.15 (0.033-0.68) p = 0.014	4.6 (1.7-12) p = 0.0021	---	---	0.11
Aggressiveness	---	---	---	2.9 (1.0-8.0) p = 0.043	0.035
Loss of appetite	---	---	4.9 (1.9-13) p = 0.0014	---	0.065
Frequent falls	---	---	2.9 (1.0-8.4) p = 0.051	---	0.025
Not being herself/himself	---	---	---	---	---
Any of the above symptoms	---	---	2.2 (1.1-4.4) p = 0.019	---	0.020
Dysuria	---	---	78 (9.5-643) p = 0.000050	---	0.38
Urinary urgency	---	---	9.4 (3.1-28) p = 0.000069	---	0.13
Urinary frequency	<10^-6^ (0-∞) p = 1.0	---	4.0 (0.97-16) p = 0.055	---	0.13

^1^Predictors in patients where a urine sample could be obtained and with information for all variables (n = 397). Forward stepwise (conditional) logistic regressions where probability for entry was 0.050 and for removal 0.10 was used. Variables that served well for the overall prediction were also kept in the model. Outcome presented as odds ratios (95% CI with p-value) for variables included in the model. Urine dipstick (nitrite positive or leukocyte esterase being 3+ or 4+), age, gender or presence of diabetes mellitus did not reach the final model for any symptom. Nagelkerke’s R-square as a measure of the model’s ability to predict presence of a symptom.

^2^With (=1) or without (=0) bacteriuria. The latter was the reference.

^3^Interleukin-6 elevated (≥5 ng/L) or not. The latter was the reference.

^4^Ongoing antibiotic treatment (n = 16) or having had antibiotics during the last month (n = 28).

^5^None of the independent variables could predict either fatigue or restlessness.

## Discussion

Recent onset of nonspecific symptoms was common among elderly residents of nursing homes. Positive urine cultures were as common in residents with as without nonspecific symptoms. Residents with positive urine cultures had higher concentrations of IL-6 in the urine. However, among residents with positive urine cultures there were no differences in IL-6 concentrations or dipstick findings between those with or without nonspecific symptoms.

### Strengths and limitations of the study

A major strength of this study is that urine specimens were collected from every participating resident capable of providing a urine sample, regardless of the presence of symptoms. Therefore, this study can compare residents having symptoms with those without symptoms.

In this study we obtained urine specimens and study protocols from 47% (421/901) of individuals registered at the nursing homes. This may appear low but is similar to previously published studies in nursing homes [3]. The main reason for not participating was substantial urinary incontinence, often combined with dementia. Twenty-five percent (222/901) refused participation. Still this may be considered acceptable when studying an elderly fragile population with a high proportion of residents with dementia as well as the ethical requirement of approval from appointed representative/relatives.

All individuals living at the nursing homes were asked to participate. Due to ethical considerations, it was not noted whether those who refused participation suffered from dementia or urinary incontinence too severe to be able to provide a urine sample. The same applied to one ward withdrawing during the ongoing study. Thus, it is assumed that some of the patients excluded, since they refused participation, would not have been eligible for this study anyway. Knowing these numbers would probably have resulted in less exclusion due to a higher number of residents not meeting the inclusion criteria.

The main focus was non-specific symptoms, and the study had enough power to suggest that IL-6 does not play a role in determining if any non-specific symptom is caused by a UTI or something else. Furthermore, these results suggest that non-specific symptoms are, in most cases, unlikely to be caused by a UTI. However, the study is underpowered to clearly sort out these issues for each specific symptom.

Residents with urinary catheters were not included in this study, therefore the results cannot be considered representative for residents with urinary catheters.

### Differentiating ABU versus UTI

It is interesting to note that a positive urine culture was not commoner among residents with nonspecific symptoms compared to residents without symptoms. There was a trend (p = 0.057) toward a lower proportion of positive urine cultures among residents with confusion occurring during the last month (Table 1). This suggests that nonspecific symptoms are not caused by bacteria in the urine. Not considering other more plausible causes of the symptoms places the patient at risk for having other undiagnosed conditions. The UTI diagnosis is all too often made in the absence of newly onset focal urinary tract symptoms.

Procedures utilizing presence of symptoms or outcomes of prior dipstick testing to influence setting of cut-off levels for CFU/mL in urine cultures to label growth as clinically significant may enhance the diagnostic procedure [36,37]. These procedures are common in microbiologic laboratories in Sweden and internationally. Using the routine clinical procedure increases clinical usefulness of the study results.

Residents with positive urine cultures had higher concentrations of IL-6 in the urine. However, among residents with positive urine cultures there were no differences in IL-6 concentrations between those with or without nonspecific symptoms. Thus IL-6 concentrations are not useful when assessing elderly residents with nonspecific symptoms and bacteria in the urine. If nonspecific symptoms are not caused by bacteria in the urine, IL-6 concentrations cannot identify a subgroup of residents with more severe inflammation in the bladder correlating to nonspecific symptoms.

There were no differences either in urine dipstick analyses for nitrite or leukocyte esterase ≥3+ between residents with positive urine cultures when comparing those with or without symptoms. Subsequently urine dipsticks are not useful when assessing elderly residents with nonspecific symptoms and bacteria in the urine.

### Antibiotic treatment and negative urine culture

Residents with recently onset confusion, loss of appetite, frequent falls and any of the nonspecific symptoms had oftener been prescribed antibiotics during the last month. This might explain the trend toward the lower prevalence of bacteriuria among residents with confusion. Also, in the logistic regressions, antibiotics during the previous month were a predictor of loss of appetite, frequent falls and “any of the nonspecific symptoms”. This supports previous studies showing that nonspecific symptoms were a common reason for suspecting UTI and the prescription of antibiotics [12-14,27]. These registered symptoms in this study might also reflect side effects of prescribed antibiotics as the elderly are more likely to retain side effects from antibiotics [38]. These residents could also represent a frailer population having more nonspecific symptoms, and also being more prone to infections, and consequently more antibiotic prescriptions.

Even if this study suggests that nonspecific symptoms are not caused by bacteria in the urine, due to the possible confounders described above, the best proof would be a future randomized controlled trial evaluating UTI antibiotic treatment of nonspecific symptoms among elderly residents of nursing homes. However, an RCT in a large population of fragile elderly individuals, many with dementia and no possibility to give statement of consent would be very difficult to carry out.

This study primarily aimed to study non UTI specific symptoms. As UTI specific symptoms were less frequent, this study was partially underpowered regarding UTI specific symptoms. However, it is interesting to note that among all symptoms urinary frequency was the only symptom where the proportion of positive urine cultures differed from those not having this symptom. Those with urinary frequency had a lower proportion of positive urine cultures and a trend (not significant) towards a higher proportion of having had antibiotic treatment during the previous month. Another explanation for this could be a shorter bladder incubation time in that group.

## Conclusions

Recently onset nonspecific symptoms were common among elderly residents of nursing homes. Residents without nonspecific symptoms had positive urine cultures as often as those with nonspecific symptoms, suggesting that nonspecific symptoms are not caused by bacteria in the urine.

Residents with positive urine cultures had higher concentrations of IL-6 in the urine. However, among residents with positive urine cultures there were no differences in IL-6 concentrations or dipstick findings between those with or without nonspecific symptoms. Thus, IL-6 concentrations in the urine and dipstick analyses are not useful when assessing elderly residents with nonspecific symptoms and bacteria in the urine.

## Competing interests

The authors declare that they have no competing interests.

## Authors’ contributions

All authors participated in the design of the study. PDS and ME carried out the data collection. PDS analysed the data and drafted the manuscript. All authors contributed to interpretation of the analyses, critical reviews and revisions, and the final approval of the paper.

## Pre-publication history

The pre-publication history for this paper can be accessed here:

http://www.biomedcentral.com/1471-2318/14/88/prepub
